# First Case of Foot Drop Associated with Capecitabine in a Patient with Thymidylate Synthase Polymorphism

**DOI:** 10.7759/cureus.995

**Published:** 2017-01-24

**Authors:** Andrew B Wilks, Muhammad W Saif

**Affiliations:** 1 Tufts Medical Center, Tufts University School of Medicine; 2 Hematology/Oncology, Tufts Medical Center, Tufts University School of Medicine

**Keywords:** capecitabine, 5-fluorouracil, dihydropyrimidine dehydrogenase (dpd), peripheral neuropathy, foot drop, thymidylate synthase (ts), pharmacogenetics

## Abstract

Capecitabine, an oral prodrug of 5-FU, has been approved by the FDA for use in patients with breast and colon cancers. In addition, capecitabine is commonly used in patients with other malignancies such as pancreatic, gastroesophageal, and hepatobiliary tract cancers. Though cerebellar toxicity is a rare but well-known side effect of intravenous 5-FU therapy, peripheral neuropathy with capecitabine has only been described in rare cases. In this case report, we describe a 79-year-old patient with locally advanced adenocarcinoma of the pancreas undergoing chemoradiation therapy with capecitabine who developed peripheral sensorimotor neuropathy. To the best of our knowledge, this is the first patient in the literature who was found to have two mutations (2R) of a 28 base-pair tandem repeat in the 5’ promoter enhancer region (5’-TSER) on both alleles (2R/2R) of thymidylate synthetase (TYMS) gene, possibly responsible for the neurotoxicity.

## Introduction

Capecitabine is a rationally designed oral formulation of fluoropyrimidine that is absorbed intact through the intestinal wall and then converted to 5-FU in three sequential enzymatic reactions, the third step being mediated by the enzyme thymidine phosphorylase (TP) at tissue level [1]. TP is known to be present in significantly higher concentrations in cancer cells than in plasma or surrounding normal tissue, thus producing a higher intratumoral concentration of 5-FU, thereby inducing a better anti-tumor effect while simultaneously sparing many of the systemic toxicities associated with 5-FU. Radiation has shown to upregulate TP, producing more 5-FU within the tumor tissue [2]. Pancreatic xenograft studies from our laboratory demonstrated a synergistic anti-tumor effect with concomitant capecitabine and radiotherapy in both radiated and contralateral lead-shielded tumors in the same animals (abscopal effects) [3]. We pioneered a phase I study, which concluded that capecitabine 800 mg/m2 BID with concurrent radiation therapy is feasible in patients with locally advanced pancreatic cancer (LAPC) [4]. Compared to intravenous 5-FU, capecitabine is associated with a lower incidence and severity of diarrhea, stomatitis, nausea, and neutropenia, but with an increased rate of hand-foot syndrome. This approach offers an easy alternative to intravenous fluorouracil as a radiosensitizer. This hypothesis was further confirmed in a phase II study by our group [5] and then all the major cooperative groups adopted capecitabine as a radiosensitizer of choice in this setting.

Peripheral neuropathy has been reported with 5-FU given alone as well as when in combination with chemotherapy agents that are known to produce cumulative peripheral neuropathy such as platinum analogs and taxanes [6]. To our knowledge, there has been only one prior report describing two patients who developed a delayed onset peripheral neuropathy following administration of capecitabine, as our group published the first two cases that developed peripheral neuropathy secondary to capecitabine (Table 1) [7]. However, in this paper, we describe the first case of foot drop associated with capecitabine in a patient with thymidylate synthase (TYMS) polymorphism. Informed consent was obtained from the patient for this study.

**Table 1:** Summary of patients who developed peripheral neuropathy following administration of 5-FU/Xeloda-based chemotherapy

Reference	Age/gender	5-FU/Xeloda (mg/m2)	Concurrent chemotherapy/radiation	Neurotoxicity	Treatment	Outcome
Stein, et al. 1998	71 male	450 i.v. push daily x 5	Levamisole	Pain in lower limbs	RTX discontinued	Discontinued Symptoms stabilized until the patient was rechallenged with 5-FU and leucovorin for liver metastasis; subsequent deterioration in neurotoxicity occurred requiring discontinuation of 5-FU/LV
Stein, et al. 1998	54 female	450 i.v. push daily x 5	Levimasole	Pain and numbness in lower limbs	RTX discontinued	Improved but incomplete resolution
Saif, et al. 2001	65 male	65 p.o. days one –three weekly x three of four weeks	Leucovorin + Eniluracil	Reduced sensation in leg leading to unsteady gait	5-FU dose reduced	Symptoms stabilized with dose reduction; gradually improved after RTX stopped with persistent foot drop
Saif, et al. 2001	70 male	23.4 p.o. days one–three weekly x three of four weeks	Leucovorin + Eniluracil	Reduced sensation in leg leading to unsteady gait	5-FU dose reduced	Symptoms stabilized with dose reduction; gradual but incomplete
Saif, et al. 2004	50 female	2500 mg/m2 in two divided doses x 14 days q21 days	None	Perioral and bilateral tingling, numbness in hands	CAP dose reduced	Symptoms resolved
Current case and Saif, et al. 2004	65 male	1600 mg/m2 in two divided doses x six weeks	Radiation (5.4 cGy)	Foot drop	CAP held; later reduced	Improved but incomplete resolution

## Case presentation

The patient was a 79-year-old Caucasian male with locally advanced adenocarcinoma of the pancreas encasing the superior mesenteric artery and celiac ganglion. His past medical history includes a hiatal hernia, varicosities in the legs, and mild hypertension. Current medications include pancrelipase, omeprazole, and lisinopril. He smoked about one pack of cigarettes per day for 40 years but stopped at the age of 55 years and denied alcohol abuse. At the initial oncologic evaluation, his physical examination did not reveal any abnormality except varicose veins on the left leg more than right. Neurological examination was also normal. He was able to ambulate without any assistance and was able to do all activities of daily living (ADLs).

**Figure 1:**
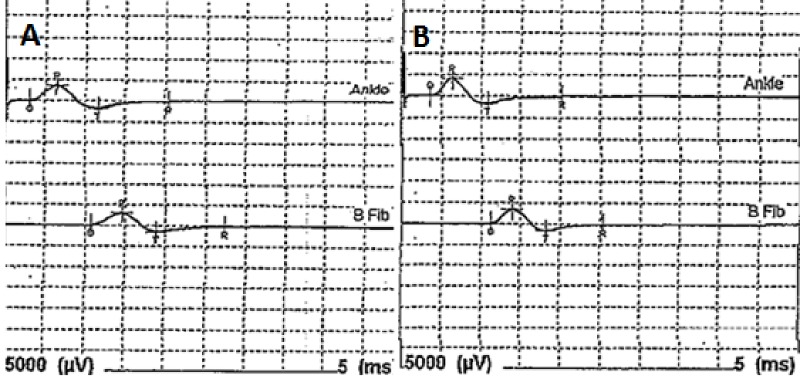
Initial EMG of bilateral peritoneal motor nerves shows peripheral nerve damage to right in comparison to left EMG of right peritoneal motor nerve (A) shows increased latency, increased duration, decreased amplitude and decreased complexity of the motor unit action potentials (MUAP) in comparison to left peritoneal motor nerve (B), indicating denervation secondary to capecitabine chemotherapy

The patient started chemoradiation therapy consisting of capecitabine (1600 mg/m2 in two divided doses = 3200 mg/m2 per day Monday to Friday with weekends off), with concurrent radiation (50.4 Gy in 28 fractions over 5.5 weeks). He tolerated the combination therapy extremely well for the first three weeks without any major toxicity except grade one nausea, grade one vomiting, grade one diarrhea and grade one hand-foot syndrome. On day three of the 4th week, he presented to the oncology clinic with new onset of gait abnormality noticed by his son that morning. The son noticed that the patient was dragging the front of his right foot on the ground when he walked. The patient further confirmed that he developed a new difficulty lifting the front part of his foot that morning when he wanted to walk to the restroom. No gross abnormal findings were elucidated on neurological examination, including cranial nerves, except unsteady gait with right-sided foot-dragging, impaired heel-toe walking, diminished sensation to pinprick in the lower extremities, and decreased distal motor strength. A magnetic resonance imaging scan of the brain and thoracolumbar spines was unremarkable. Laboratory studies including complete blood count, chemistry, liver function tests, vitamin B12, thyroid stimulating hormone (TSH) test, rheumatoid factor, thiamine levels, antibodies against double-stranded DNA phospholipid and cardiolipin were collected and all came within normal limits. The patient was not receiving any other potentially neurotoxic medications. Nerve conduction studies and electromyogram revealed acute, axonal sensorimotor polyneuropathy with secondary demyelinative features, with an increasing proximal to distal neuropathic gradient (Figure 1).

We immediately halted administration of capecitabine, consulted neurology as well as physical therapy. Neurology agreed with our assessment and suggested not to resume the drug as no other etiology was found. They suggested to rule out cancer progression as sometimes these neurological abnormalities might be manifesting paraneoplastic syndrome and the repeat CT imaging of scan of the abdomen, pelvis, and chest showed a slight improvement in tumor size in the pancreas and no distant metastasis.

In addition to above tests, we also performed genetic studies related to capecitabine including dihydropyrimidine dehydrogenase (DPD) deficiency and thymidylate synthase (TYMS) polymorphism. DPD gene mutation analysis (Quest Diagnostics Nichols Institute-San Juan Capistrano, CA) was negative for the IVS14+1G>A mutation in the DPD gene, which accounts for 50% of the DPD deficiency alleles. However, the patient was found to have two mutations (2R) of a 28 base-pair tandem repeat in the 5’ promoter enhancer region (5’-TSER) on both alleles (2R/2R) of TYMS gene. This 2R/2R genotype predicts low TYMS expression.

**Figure 2:**
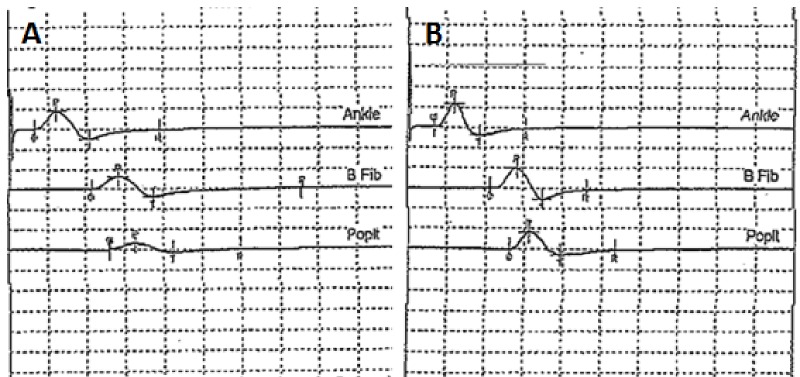
EMG of peritoneal motor nerves at seven months shows ongoing peripheral nerve damage to right in comparison to left EMG of right peritoneal nerve (A) shows decreased amplitude and decreased complexity of MUAP in comparison to left peritoneal motor nerve (B), indicating ongoing peripheral nerve damage months after cessation of capecitabine chemotherapy

The patient’s symptoms slightly improved within the next four weeks after holding capecitabine and initiation of physical therapy. However, after one week of delay, he finished the remaining fractions of radiation therapy, impressed by the CT scan findings showing shrinkage of the tumor. After a short break of two weeks, he came back to the oncology clinic for further discussion on treatment. After extensive discussion, the decision was made to proceed with a different chemotherapy and hence single agent gemcitabine 1000 mg/m2 weekly x three out of four weeks was administered starting after another two weeks delay. At that time, he had gradual improvement in his gait (using a cane), balance and coordination. He received a total of six cycles (four weeks = one cycle) of gemcitabine with no new neurological deficit. Physical examination of seven months after discontinuing revealed residual grade two sensory loss in the feet and grade one motor loss with dorsiflexion. Repeat electromyogram (EMG) test at that time (Figure 2) showed chronic peripheral nerve degeneration, despite the improvement of symptoms.

## Discussion

The 5-fluorouracil (5-FU) and its prodrug capecitabine are the third most commonly used agents in oncology for the treatment of various cancers. The majority of the randomized phase III studies have shown comparable end results when comparing capecitabine and 5-FU; though capecitabine offers the advantages of oral convenience and less bone marrow suppression. The most common side effects of capecitabine include diarrhea, neutropenia, and alopecia although capecitabine (CAP) causes more cases of severe hand-foot syndrome. Capecitabine is metabolized to 5-FU via a three-step enzymatic process, the last step catalyzed by TP [1,5]. In addition, TP is also involved in the activation of 5-FU into fluorodeoxyuridine that will further inhibit the DNA synthesis. Tumor concentration of TP is three-10 times higher than the healthy tissue. This can enable selective drug activation of 5-FU at the tumor site and possibly decrease systemic toxicity [8].

In addition to TP, other genes, such as TYMS, are considered as potential factors for 5-FU toxicity and efficacy. TYMS is strongly inhibited by 5-FU and considered to be the major drug target. TYMS catalyzes the intracellular conversion of deoxyuridylate to deoxythymidylate that is the sole de novo source of thymidylate, an essential precursor for DNA synthesis. The active metabolite of 5-FU, 5-fluorodeoxyuridylate (5FdUMP), binds to TYMS and inhibits it by forming a stable tertiary complex. The human TYMS is polymorphic with either double or triple tandem, repeats of a 28 base-pair sequence downstream of the cap-site in the 59-terminal regulatory region. The TYMS genotype predicts TYMS mRNA expression in metastasized colon tumors and normal liver tissue as well as predicts response and toxicity to 5-FU [9].

Finally, 5-FU is catabolized into dihydrofluorouracil by the DPD enzyme that is present in almost all tissues. DPD is the initial rate-limiting enzyme in the catabolism of 5-FU. Polymorphic abnormality of DPYD gene is one of the most well known pharmacogenetic syndromes in medical oncology at present [8]. Patients with DPD deficiency experience profound systemic toxicity following the administration of 5-FU or capecitabine presumably secondary to prolonged exposure to 5-FU due to decreased drug catabolism.

Capecitabine, an oral prodrug of 5-FU, has been found in higher concentrations in cancer cells when administered concurrently with radiation therapy. This is due to a higher concentration of TP, the final of three proteins, which convert capecitabine to 5-FU, in cells damaged by radiation [1,4]. One can argue that uncommon toxicities may occur due to this synergy as we have previously published [10]. Our patient was found to have two mutations (2R) of a 28 base-pair tandem repeat in the 5’ promoter enhancer region (5’-TSER) on both alleles (2R/2R) of TYMS gene. This 2R/2R genotype predicts low TYMS expression. While controversy exists in the literature, overall this finding predicts improved survival of patients with colorectal adenocarcinoma receiving 5-FU chemotherapy but also increased the risk for 5-FU toxicity. Our patient was found to have no abnormalities of DPD. This report is the first in medical literature associating peripheral neuropathy to TYMS abnormality in a patient receiving capecitabine. It has been published prior that capecitabine crosses the blood-brain barrier, though the biochemical basis of neurological toxicities has not been elucidated.

Neurological side effects have been reported on rare occasions and should be borne in mind while administrating this class of drugs to patients. Dose adjustment and discontinuation must be assessed in any patient developing side effects to 5-FU therapies.

## Conclusions

In this case report, we describe a 79-year-old patient with locally advanced adenocarcinoma of the pancreas, undergoing chemoradiation therapy with capecitabine who developed peripheral sensorimotor neuropathy. Peripheral sensory motor conduction studies confirmed peripheral neuropathy. Pharmacogenetic studies related to genes associated with 5-FU metabolism showed polymorphism of TS, possibly causing the nerve damage in this patient. Regardless, dose reduction or discontinuation of capecitabine therapy is indicated in any patient who develops these complications.
